# Vasohibin‐1 deficiency enhances renal fibrosis and inflammation after unilateral ureteral obstruction

**DOI:** 10.14814/phy2.12054

**Published:** 2014-06-27

**Authors:** Hiroyuki Watatani, Yohei Maeshima, Norikazu Hinamoto, Hiroko Yamasaki, Haruyo Ujike, Katsuyuki Tanabe, Hitoshi Sugiyama, Fumio Otsuka, Yasufumi Sato, Hirofumi Makino

**Affiliations:** 1 Department of Medicine and Clinical Science, Okayama University Graduate School of Medicine, Dentistry and Pharmaceutical Sciences, Okayama, 700‐8558, Japan; 2 Department of Chronic Kidney Disease and Cardiovascular Disease, Okayama University Graduate School of Medicine, Dentistry and Pharmaceutical Sciences, Okayama, 700‐8558, Japan; 3 Center for Chronic Kidney Disease and Peritoneal Dialysis, Okayama University Graduate School of Medicine, Dentistry and Pharmaceutical Sciences, Okayama, 700‐8558, Japan; 4 Department of Vascular Biology, Institute of Development, Aging, and Cancer, Tohoku University, Sendai, Japan

**Keywords:** Inflammation, macrophage, TGF‐*β*1, tubulointerstitial fibrosis, unilateral ureteral obstruction, vasohibin‐1

## Abstract

Tubulointerstitial injuries are known to predict the deterioration of renal function in chronic kidney disease (CKD). We recently reported the protective role of Vasohibin‐1(VASH‐1), a negative feedback regulator of angiogenesis, in diabetic nephropathy, but its impact on tubulointerstitial injuries remains to be elucidated. In the present study, we evaluated the role of endogenous VASH‐1 in regulating the tubulointerstitial alterations induced by unilateral ureteral obstruction (UUO), and assessed its role on fibrogenesis and the activation of Smad3 signaling in renal fibroblasts. UUO was induced in female Vasohibin‐1 heterozygous knockout mice (VASH‐1^+/−^) or wild‐type (WT) (VASH‐1^+/+^) littermates. Mice were sacrificed on Day 7 after left ureter ligation, and the kidney tissue was obtained. Interstitial fibrosis, the accumulation of type I and type III collagen and monocytes/macrophages infiltration in the obstructed kidneys (OBK) were significantly exacerbated in VASH‐1^+/−^ mice compared with WT mice (Day 7). The increases in the renal levels of TGF‐*β*1, pSmad3, NF‐*κ*B pp65, CCL2 mRNA, and the number of interstitial fibroblast‐specific protein‐1 (FSP‐1)^+^ fibroblasts in the OBK were significantly aggravated in VASH‐1^+/−^ mice. In addition, treatment with VASH‐1 siRNA enhanced the TGF‐*β*1‐induced phosphorylation of Smad3, the transcriptional activation of the Smad3 pathway and the production of type I/type III collagen in fibroblasts, in vitro. Taken together, our findings demonstrate a protective role for endogenous VASH‐1 on tubulointerstitial alterations via its regulation of inflammation and fibrosis and also show the direct anti‐fibrotic effects of VASH‐1 on renal fibroblasts through its modulation of TGF‐*β*1 signaling.

## Abstract (precis)

In the present study, we evaluated the role of endogenous Vasohibn‐1 VASH‐1 in regulating tubulointerstitial alterations induced by unilateral ureteral obstruction (UUO), and assessed its regulatory role on fibrogenesis and the activation of TGF‐beta/Smad3 signaling in renal fibroblasts. This is the first study to demonstrate the potential protective role for endogenous VASH‐1 on tubulointerstitial alterations via regulating inflammation and fibrosis, partly mediated via its direct anti‐fibrotic effects on renal fibroblasts through modulating TGF‐*β*1 signaling.

## Introduction

Tubulointerstitial alterations are characteristic histological features involved in the progression of chronic kidney disease (CKD). These alterations include the interstitial infiltration of monocytes/macrophages, accumulation of myofibroblasts, proliferation of interstitial fibroblasts and accumulation of extracellular matrix (ECM) proteins, resulting in interstitial fibrosis (Eddy 1996).

Unilateral ureteral obstruction (UUO) is a known experimental model developing renal tubulointerstitial injuries. Various factors including profibrotic transforming growth factor (TGF)‐*β*1 (Lan et al. 2003) and cytokines/chemokines, such as IL‐6 and CCL2, are involved in the process of UUO (Schreiner et al. 1988).

Myofibroblasts play an important role in the process of kidney fibrosis, including that occurring during UUO, but the origin of the myofibroblasts associated with renal interstitial fibrosis has been debated. The proliferation of residential tissue fibroblasts, the differentiation of bone marrow‐derived mesenchymal stem cells, tubular epithelial cells, vascular pericytes of the peritubular capillaries (PTC; Lin et al. 2011) and endothelial cells are the postulated origins of the renal myofibroblasts (LeBleu et al. 2013).

The transcription factor NF‐*κ*B plays an important role in inflammation and immune responses. In mammalian cells, classical NF‐*κ*B is composed of a heterodimer of the p50 and p65/RelA proteins, and the p65/RelA subunit possesses transactivational activity (Ghosh et al. 1998; Ghosh and Karin 2002). NF‐*κ*B activation is associated with the pathogenesis of CKD including UUO (Chung et al. 2009), atherosclerosis, and diabetes (Saito et al. 2011).

Transforming growth factor‐*β*/Smad3 signaling plays a critical role in promoting renal fibrosis (Bottinger and Bitzer 2002; Schnaper et al. 2002; Wang et al. 2005b; Liu 2006). TGF‐*β*1 signals through the heteromeric complex of TGF‐*β* type I and type II receptors to activate Smad2 and Smad3. In particular, the activation of Smad3 plays an important role in various processes including cell growth, differentiation, apoptosis, and tissue repair (Yang et al. 1999; Roberts et al. 2003; Flanders 2004), and Smad3 serves as a key mediator of the TGF‐*β* signaling leading to renal fibrosis (Sato et al. 2003).

Vasohibin‐1 (VASH‐1) was identified from a microarray analysis searching genes upregulated by VEGF‐A in endothelial cells (Watanabe et al. 2004). VASH‐1 regulates the proliferation and migration of endothelial cells, and thus serves as a negative feedback regulator of angiogenesis. A critical role of VASH‐1 in the maintenance of endothelial cells against cellular stressors, and the posttranscriptional regulation mediated by the binding of HuR proteins to AU‐rich elements in the 3′ untranslated region of VASH‐1 mRNAs have been reported (Miyashita et al. 2012). To date, cell surface receptors for VASH‐1 have not been reported. However, therapeutic effects of VASH‐1 on tumor growth, proliferative retinopathy and atherosclerosis models have been reported (Watanabe et al. 2004; Shen et al. 2006; Yamashita et al. 2006). We previously reported the therapeutic effects of the adenoviral transfer of VASH‐1 (AdhVASH‐1) in mouse diabetic nephropathy models, and showed direct effects of VASH‐1 on glomerular mesangial, endothelial cells as well as podocytes (Nasu et al. 2009; Saito et al. 2011).

Although we previously demonstrated the inhibitory effects of AdhVASH‐1 on mesangial matrix expansion in diabetic animals (Nasu et al. 2009; Saito et al. 2011), the pathophysiological role of VASH‐1 in renal interstitial fibrosis remains unclear. Thus, in the present study, we investigated the functional role of endogenous VASH‐1 in regulating the inflammation and renal fibrosis in a mouse model of UUO using VASH‐1 deficient mice. In addition, the mechanisms responsible for the effects of VASH‐1 on fibrogenesis and Smad3 signaling were also examined in vitro.

## Materials and Methods

### Animals and experimental design

The experimental protocol was approved by the Animal Ethics Review Committee of Okayama University Graduate School of Medicine, Dentistry, and Pharmaceutical Sciences, Okayama, Japan.

Female mice between nine and 11 weeks of age were used for the studies. Homozygous VASH‐1 gene knockout (KO) mice on a C57BL/6J background were generated by gene targeting, as described previously (Kimura et al. 2009). Wild‐type (WT) C57BL/6J mice served as controls.

Unilateral ureteral obstruction was induced in female Vasohibin‐1 heterozygous knockout mice (VASH‐1^+/−^) or their WT (VASH‐1^+/+^) littermates. The left ureter of the mice was exposed through a mid‐abdominal incision, and was ligated at two positions ~1 cm below the renal hilum with 3–0 silk under pentobarbital anesthesia, as previously described (Satoh et al. 2001; Sunami et al. 2004; Nasu et al. 2012). Ureters of sham‐operated mice were manipulated, but not ligated. Weight‐matched (20–22 g) mice were divided into four subgroups: (i) WT sham‐operated, (ii) WT UUO, (iii) VASH‐1^+/−^ sham‐operated and (iv) VASH‐1^+/−^ UUO (*N *=**5 for each subgroup). All mice were sacrificed on Day 7 after left ureter ligation, and the bilateral kidneys and serum samples were collected.

### Blood examination

The concentration of blood urea nitrogen (BUN) and serum creatinine were measured at SRL (Okayama, Japan). The serum creatinine levels were measured by the enzymatic colorimetric method as described previously (Hashimoto et al. 2004).

### Histological analysis

Seven days following the induction of UUO, the kidneys were obtained, fixed in 10% buffered formalin, and embedded in paraffin. Sections (4 *μ*m thick) were stained with Masson's Trichrome and observed under light microscopy. Interstitial fibrotic areas with blue staining were evaluated by a computer image analysis using the Lumina Vision software program (Mitani, Fukui, Japan). Fifteen consecutive fields of the renal cortex were randomly chosen and observed at 400× magnification. Tubular cells, tubular lumens, glomeruli, and blood vessels were excluded from evaluation. This fraction represented the relative renal interstitial volume and was expressed as a percentage of the measured area (Satoh et al. 2001; Nasu et al. 2012). The histological evaluation was performed in a blinded manner by two investigators, and the scores were averaged.

### Sirius red staining

Collagen deposition was investigated by picro‐Sirius red staining. The 4 *μ*m paraffin sections were deparaffinized and rehydrated. Nuclei were stained with Weigert's hematoxylin for 20 minutes at room temperature. After being washed, the sections were stained with 0.1% Sirius Red F3BA (Direct Red 80; Sigma‐Aldrich, St.Louis, MO) in a saturated picric acid solution for 1 h and were washed in two changes of acidified water. After being washed, the slides were dehydrated and mounted. Red‐stained interstitial fibrotic areas were assessed by a computer image analysis using the Lumina Vision software program. Fifteen fields of the renal cortex were randomly selected at 400× magnification and glomeruli and blood vessels were excluded. The collagen staining was expressed as a percentage of the measured area, which represented the interstitial space and was determined as the relative volume of the interstitium. A histological evaluation was performed in a blinded fashion by two investigators, and the scores were averaged.

### Immunohistochemistry

The immunohistochemical analyses were performed using 4 *μ*m frozen sections as described previously (Maeshima et al. 1998; Ichinose et al. 2006; Tanabe et al. 2007; Nasu et al. 2009, 2012). Briefly, sections were incubated with polyclonal rabbit an anti‐human fibroblast‐specific protein‐1 (FSP‐1; S100A4) antibody (1:100 dilution, A5114; Dako Cytomation, Carpinteria, CA) or rat anti‐mouse F4/80 antibody (1:200 dilution, A3–1; Serotec, Oxford, UK) and exposed to secondary antibodies, Immun‐Star goat anti‐rabbit (GAR)‐horseradish peroxidase (HRP) conjugate (1:200 dilution, 170–5046; Bio‐Rad, Hercules, CA), or HRP‐labeled goat anti‐rat IgG (Millipore, Billerica, MA). Diaminobenzidine was used as a chromogen. All slides were counterstained with hematoxylin.

The renal interstitial monocyte/macrophage infiltration was evaluated by the number of F4/80^+^ cells in the renal cortex. The F4/80^+^ cellularity was determined in 15 randomly selected non‐overlapping fields (magnification, ×400) in each section of the individual mouse renal cortex. The average number of F4/80^+^ cells from five separate animals was determined (Satoh et al. 2001; Nasu et al. 2012). Similarly, the number of interstitial FSP‐1^+^ cells was determined (magnification, ×400).

### Immunofluorescence

Immunofluorescent staining was performed using 4 *μ*m frozen sections as described previously to assess the interstitial accumulation of types I and III collagen (Ichinose et al. 2005; Tanabe et al. 2007; Nasu et al. 2009, 2012). Sections were blocked with 10% normal goat serum (426041; Nichirei Bioscience Inc., Tokyo, Japan) followed by incubation with primary antibodies, polyclonal rabbit anti‐mouse type I collagen antibody (1:100 dilution, AB765‐P; Millipore), polyclonal rabbit anti‐mouse type III collagen antibody (1:200 dilution, LB‐1393; Cosmo Bio, Tokyo, Japan) and polyclonal rabbit anti‐mouse vasohibin‐1 antibody (1:400 dilution) for 1 h. Subsequently, the sections were incubated with Alexa Fluor 488‐labeled goat anti‐rabbit IgG (1:100 dilution, A11008; Invitrogen, Carlsbad, CA) for 1 h. Sections were observed using a fluorescence microscope (BZ‐Analyzer; Keyence, Osaka, Japan) and images were obtained. Normal rabbit IgG was used as a negative control. To assess the type I and type III collagen‐positive areas, the image files (1392 × 1040 pixels) at 400× magnification were analyzed using Lumina Vision software.

To assess the interstitial accumulation of *α*SMA^+^ myofibroblasts, 4 *μ*m frozen sections were blocked with a protein‐blocking solution (Protein Block Serum‐Free, X0909; Dako Denmark A/S, Glostrup, Denmark), followed by incubation with a monoclonal mouse anti‐human *α*SMA antibody (1:800 dilution, A2547; SIGMA, Sigma‐Aldrich) for 30 min. Subsequently, the sections were incubated with Alexa Fluor 488‐labeled goat anti‐mouse IgG (1:100 dilution, A11001; Invitrogen) for 15 min. To evaluate the interstitial *α*SMA^+^ area, color image files were obtained as TIFF files and were analyzed by using the Lumina Vision software program. In each kidney, 15 random measurements of the renal cortex were performed at a magnification of 200×, and blood vessels were excluded. The image files were opened in the grayscale mode. The interstitial *α*SMA^+^ area was calculated using the following formula: [{*X* (density) × positive area (*μ*m^2^)}/tubulointerstitial total area (*μ*m^2^)], where the staining density is indicated by a number from 0 to 256 in the grayscale.

### Western blot analysis

The Western blot analyses were performed as described previously (Maeshima et al. 2002; Ichinose et al. 2005; Tanabe et al. 2007; Nasu et al. 2009, 2012; Saito et al. 2011). Briefly, the renal cortex was homogenized in radioimmunoprecipitation (RIPA) lysis buffer (sc‐24948; Santa Cruz Biotechnology, Dallas, TX) at 4°C. After centrifugation at 20,817 g for 30 min at 4°C, the supernatant was collected and stored at −80°C until use. The total protein concentration was measured using the DC‐protein determination system (Bio‐rad) using bovine serum albumin (BSA) as a standard. Samples were processed for SDS‐PAGE, and proteins were electrotransferred onto a nitrocellulose membrane (iBlot Gel transfer stacks, IB3010‐02; Invitrogen). The membranes were blocked with 5% nonfat dry milk in 1× Tris‐buffered saline containing 0.1% Tween‐20 for 1 h, incubated overnight with polyclonal rabbit anti‐TGF‐*β*1/2/3 (1:500 dilution, sc‐7892; Santa Cruz), monoclonal rabbit anti‐Smad3 (1:1000 dilution, #9523; Cell Signaling, Danvers, MA), monoclonal rabbit anti‐phospho‐Smad3(Ser423/425) (1:1000 dilution, #9520; Cell Signaling), polyclonal rabbit anti‐NF‐*κ*B p65 (1:1000 dilution, #3034; Cell Signaling), polyclonal rabbit anti‐phospho‐NF‐*κ*B p65 (1:1000 dilution, #3031; Cell Signaling), polyclonal rabbit anti‐I*κ*B*α* (1:1000 dilution, sc‐371; Santa Cruz), polyclonal rabbit anti‐phospho‐I*κ*B*α* (1:1000 dilution, #2859; Cell Signaling), or polyclonal rabbit anti‐VEGF‐A (1:1000 dilution, A‐20 sc‐152; Santa Cruz) antibodies at 4°C. After incubation with horseradish peroxidase‐labeled secondary antibodies; Immun‐Star GAR‐HRP Conjugate (1:3000 dilution, 170‐5046; Bio‐rad) for 1 h, the signals were detected using an enhanced chemiluminescence system (Amersham, Piscataway, NJ). The membranes were reprobed with rabbit anti‐actin antibodies (1:5000 dilution, ab8227; Abcam, Cambridge, MA) to serve as controls for equal loading. The density of each band was determined using the NIH Image J software program (Wayne Rashand, Bethesda, MD), and was expressed as a value relative to the density of the corresponding band obtained from the actin immunoblot.

### Cell culture

NRK‐49F (09‐1570; DS pharma biomedical, Osaka, Japan), a normal rat kidney fibroblast cell line was cultured in Dulbecco's modified Eagle's medium (Sigma Aldrich) supplemented with 5% fetal bovine serum (FBS), penicillin G (100 U/mL), and 100 *μ*g/mL of streptomycin on T75 flasks at 37°C in 5% CO_2_. The cells were seeded at approximately 70% confluence in complete medium containing 5% FBS for 24  h at 37°C. Cells were used between passages 10 and 15.

### Gene silencing by stealth siRNA

NRK‐49F cells were transfected with small interfering RNA using the Stealth Select RNAi (Invitrogen) according to the manufacturer's instructions. The nucleotide sequences of the stealth siRNAs used in this study were as follows: for rat VASH‐1, 5′‐GGGCUGAUGGAUCUGGCCAAGGAAA‐3′ (forward) and 5′‐UUUCCUUGGCCAGAUCCAUCAGCCC‐3′ (reverse). Briefly, cells were precultured in DMEM containing 10% FBS in the absence of antibiotics for 24 h. The cells (5.0 × 10^3^) were then transfected with 60 pmol of siRNA (Primer Number: 156549, Invitrogen) and Lipofectamine RNAi MAX (Invitrogen) at a final siRNA concentration of 20 nmol/L. At 12 h post‐transfection, the medium was replaced with fresh DMEM containing 0.5% FBS, and the cells were then incubated for an additional 24 h. Then, the cells were treated with TGF‐*β*1 (240‐B; R&D Systems, Minneapolis, MN) in fresh DMEM containing 0.5% FBS for 24 h. After being washed, the cells were lysed, and RNA was extracted with the TRIzol reagent.

Specific gene silencing was verified by RT‐PCR and a Western blot analysis, and this protocol resulted in optimal knockdown efficiency. Scrambled siRNA‐transfected controls were included in all experiments. A similar method was used for VASH1 siRNA transfection for the luciferase assay.

### Transient transfection and luciferase assay

NRK‐49F cells (3.0 × 10^4^ cells) were cultured in DMEM containing 10% FBS for 24 h in the absence of antibiotics. After transfection of siRNA for 8 h, the medium was replaced with fresh DMEM containing 5% FBS and antibiotics. The cells were then transiently transfected with 500 ng of 3TP–Luc reporter plasmid and 50 ng of cytomegalovirus‐*β*‐galactosidase plasmid (pCMV‐*β*‐gal) using X‐treme GENE 9 (Roche Diagnostics GmbH, Mannheim, Germany). At 12 h post‐transfection, the cells were treated with TGF‐*β*1 in fresh DMEM containing 0.5% FBS for 24 h. After being washed, the cells were lysed with Cell Culture Lysis Reagent (Luciferase Assay Systems Kit E4030; Promega, Madison, WI). The Luciferase activity and *β*‐galactosidase (*β*‐gal) activity of the cell lysates were measured by a luminometer (AB‐2200‐R, ATTO, Tokyo, Japan). The data are shown as the ratio of luciferase to the *β*‐gal activity.

### RNA extraction and quantitative real‐time PCR

Total RNA was extracted from the kidneys of each mouse or from NRK‐49F cells using the RNeasy Kit (Qiagen, Chatsworth, CA) and was stored at −80°C until use. The total RNA was subjected to RT using a first‐strand cDNA synthesis system (Invitrogen) with random hexamers and reverse transcriptase. Quantitative real‐time PCR was then carried out using primers for mouse VASH‐1, mouse TGF‐*β*1, mouse CCL‐2(MCP‐1), mouse CD11c, mouse IL‐10, mouse CD206, rat VASH‐1, rat collagen 1*α*_1_ chain (Col1A1), rat collagen 3*α*_1_ chain (Col3A1), and GAPDH (internal control). The cDNA was diluted 1:10 with autoclaved deionized water. For the detection of the mRNA levels, 5 *μ*L of the diluted cDNA was added to the Lightcycler mastermix, which included 0.5 *μ*mol/L of specific primers, 3 mmol/L of MgCl_2_ and 2 *μ*L of Master SYBR Green. To detect the level of GAPDH, 5 *μ*L of the diluted cDNA was added to the Lightcycler mastermix, which contained 0.2 *μ*mol/L of specific primers, 3 mmol/L of MgCl_2_, and 2 *μ*L of SYBR Premix Ex Taq (Takara Bio, Shiga, Japan). These reaction mixtures were filled to a final volume of 20 *μ*L with water. The PCR reactions were carried out in a real‐time PCR cycler (Lightcycler; Roche Diagnostics). The program was optimized and finally performed as denaturation at 95°C for 2 min followed by 40 cycles of amplification (each mRNA; 95°C for 30 s, 60°C for 10 s, and 72°C for 8 s). The temperature ramp rate was 20°C/s. At the end of each extension step, the fluorescence was measured to quantitate the PCR products. After completion of the PCR, the melting curve of the product was measured by a temperature gradient from 65 to 95°C at 0.1 or 0.2°C/s with continuous fluorescence monitoring to produce a melting profile of the primers. The amount of PCR products was normalized to the level of GAPDH to determine the relative expression ratio for each mRNA.

The oligonucleotide primers specific for mouse VASH‐1, mouse TGF‐*β*1, mouse CCL‐2 (MCP‐1), mouse CD11c, mouse IL‐10, mouse CD206, rat VASH‐1, rat Col1A1, rat Col3A1, and GAPDH are detailed in Table 1. Four independent experiments were performed.

**Table 1.** The primers used for real‐time PCR

mRNA	Strand	Sequence
Mouse		
VASH‐1	forward	5‐AGCACAGAGAGATGAAGGAAC‐3
	reverse	5‐CGTCGTCGGCTGGAAAGTAG‐3
TGF‐*β*_1_	forward	5‐GTGTGGAGCAACATGTGGAACTCTA‐3
	reverse	5‐TTGGTTCAGCCACTGCCGTA‐3
MCP‐1/CCL2	forward	5‐AGGTCCCTGTCATGCTTCT‐3
	reverse	5‐CTGCTGGTGATCCTCTTGT‐3
CD11c	forward	5‐AGGTCTGCTGCTGCTGGCTA‐3
	reverse	5‐GGTCCCGTCTGAGACAAACTG‐3
IL‐10	forward	5‐GACCAGCTGGACAACATACTGCTAA‐3
	reverse	5‐GATAAGGCTTGGCAACCCAAGTAA‐3
CD206	forward	5‐TCGAGACTGCTGCTGAGTCCA‐3
	reverse	5‐AGACAGGATTGTCGTTCAACCAAAG‐3
GAPDH	forward	5‐TGTGTCCGTCGTGGATCTGA‐3
	reverse	5‐TTGCTGTTGAAGTCGCAGGAG‐3
Rat		
VASH‐1	forward	5‐AGCCCTCATCGCAGGAACA‐3
	reverse	5‐CCTCACACCCGGATCTGGTA‐3
collagen 1*α*_1_ chain (Col1A1)	forward	5‐CCAGCAAAGGCAATGCTGAA‐3
	reverse	5‐TTGTTGCAGAGGCCATGGAG‐3
collagen 3*α*_1_ chain (Col3A1)	forward	5‐CGGAAGCACTGGTGGACAGA‐3
	reverse	5‐GAAGGCCAGCTGTACATCAAGGA‐3
GAPDH	forward	5‐GGCACAGTCAAGGCTGAGAATG‐3
	reverse	5‐ATGGTGGTGAAGACGCCAGTA‐3

### Statistical analysis

All values are expressed as the means ± SEM. Results were analyzed using one‐way ANOVA followed by Tukey's post hoc test when the data were parametric. If necessary, the data were analyzed with the nonparametric Kruskal–Wallis test followed by Steel‐Dwass multiple comparisons test. A level of *P *<**0.05 was considered statistically significant.

## Results

### Body weight, kidney weight, renal function, and characterization of heterozygous VASH‐1 knockout mice

**Figure 1.**
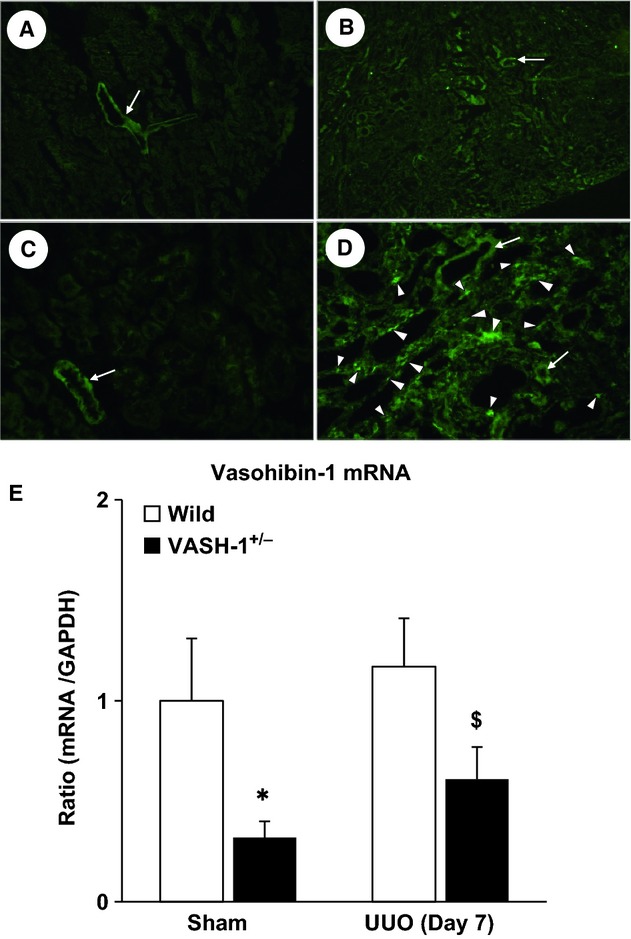
Characterization of the VASH‐1 KO mice. (A–D) The VASH‐1 antibody was used to stain serial kidney sections from sham‐operated wild‐type (WT) (A, C) and WT/unilateral ureteral obstruction (UUO) (B, D) mice. (A, B) Original magnification; ×80. (C, D) Original magnification; ×400. (A, C) The immunoreactivity for VASH‐1 was mainly observed in the blood vessels (arrows). (B, D) The immunoreactivity for VASH‐1 was observed mainly in the renal interstitial cells (arrowheads), but was also observed in the blood vessels (arrows). (E) Real‐time PCR of WT (VASH1^+/+^) and VASH1^+/−^ kidneys. The levels of VASH‐1 mRNA were normalized to those of GAPDH. The VASH‐1 mRNA levels were decreased in the VASH1^+/−^ sham‐operated mice compared with the WT sham‐operated control mice. Similarly, the levels of VASH‐1 mRNA were decreased in the OBK of the VASH‐1^+/−^ UUO mice compared with the WT UUO mice. OBK, obstructed kidneys.

The body weight was not significantly different among the 4 experimental groups. The weight of the obstructed kidneys (OBK) of the WT animals on Day 7 was significantly higher than that of sham‐operated WT mice. The weight of the OBK of VASH‐1^+/−^ animals was significantly lower than that of the WT‐UUO group (Table 2). The serum creatinine and BUN levels were not significantly different among the 4 experimental groups (data not shown). Immunofluorescent staining revealed that VASH‐1 was distributed in the blood vessels in the control kidneys (Fig. 1A and C). Increased immunoreactivity for VASH‐1 was observed in the OBK in the WT mice, mainly in the interstitial area, presumably in inflammatory cells or fibroblasts, as well as in the blood vessels (Fig. 1B and D). As shown in Figure 1E, the mRNA levels of VASH‐1 in the kidneys were significantly lower in the VASH‐1^+/−^ animals compared with the WT animals in both the sham and UUO groups.

**Table 2.** The kidney/body weight ratios of mice with unilateral ureteral obstruction

	Sham	Day 3	Day 7
Wild‐type			
Left (ligated)	5.68 ± 0.31	6.93 ± 0.14	6.98 ± 0.31#
Right (contralateral)	5.92 ± 0.11	6.27 ± 0.08	5.99 ± 0.04
VASH‐1^+/−^			
Left (ligated)	5.58 ± 0.18	7.23 ± 0.33†	6.06 ± 0.44*$
Right (contralateral)	5.81 ± 0.24	5.98 ± 0.33	5.7 6 ± 0.27

The values are the means ± SE of five animals/group.

* *P *<**0.05 versus wild‐type left (ligated) on Day 7.

# *P *<**0.05 versus wild‐type right (contralateral) on Day 7.

$ *P *<**0.05 versus VASH^+/−^ left (ligated) at Day 3.

† *P *<**0.05 versus VASH^+/−^ right (contralateral) on Day 3.

### Histology and morphometric analysis

**Figure 2.**
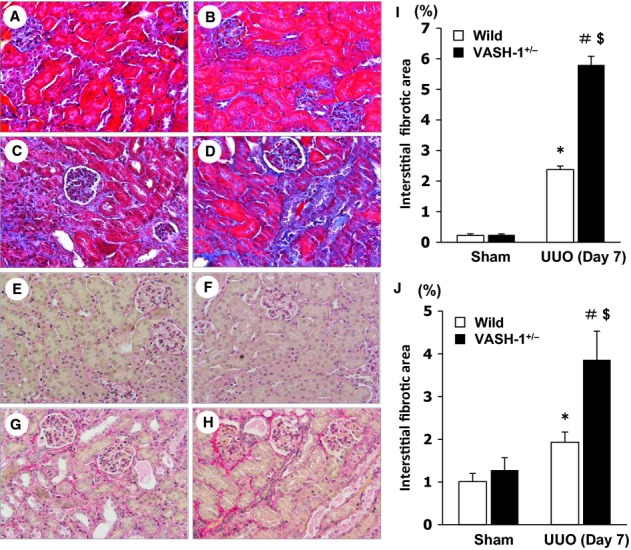
Accelerated tubulointerstitial fibrosis in the VASH‐1^+/−^ unilateral ureteral obstruction (UUO) mice. (A–D) Representative light microscopic findings of the renal cortex of the obstructed kidneys (OBK) on Day 7 (Masson's Trichrome staining, original magnification; ×400) for wild‐type (WT) sham‐operated (A), VASH1^+/−^ sham‐operated (B), WT UUO (C) and VASH1^+/−^ UUO (D) mice. (I) The interstitial fibrosis in the OBK was significantly exacerbated in the VASH1^+/−^ UUO compared with the WT UUO mice. (E–H) Representative light microscopic findings of the renal cortex of the OBK on Day 7 (Sirius red staining, original magnification; ×400) for WT sham‐operated (E), VASH1^+/−^ sham‐operated (F), WT UUO (G) and VASH1^+/−^ UUO (H) mice. (J) The interstitial fibrosis in the OBK was significantly exacerbated in the VASH1^+/−^ UUO compared with WT UUO mice. *n *=**5 for each group. *P <**0.05 versus WT sham. ^#^*P *<**0.05 versus VASH1^+/−^ sham. ^$^*P *<**0.05 versus WT UUO. Each column shows the means ± SEM.

The morphological alterations of kidney tissue were analyzed by Masson's Trichrome staining and Sirius red staining. Sham‐operated VASH‐1^+/−^ mice did not exhibit any histological alterations. Tubular dilation, atrophy, interstitial infiltration of mononuclear cells, and interstitial fibrosis were observed in the OBK on Day 7 in the WT‐UUO group (Fig. 2C and G). These histological alterations in the OBK were markedly exacerbated in the VASH‐1^+/−^ UUO group (Fig. 2D and H), which was confirmed by the quantitative analysis (Fig. 2I and J). There were no evident morphological alterations in the glomeruli or intrarenal blood vessels in the OBK of any experimental groups.

### Immunohistochemical evaluation of interstitial type I and type III collagens

**Figure 3.**
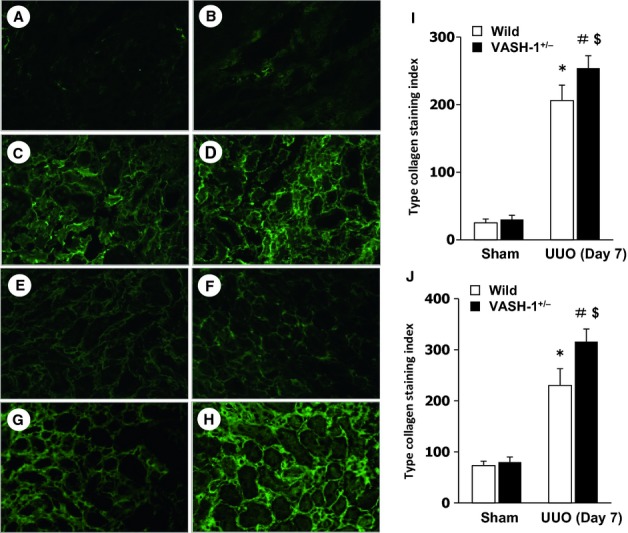
Immunofluorescent staining of type I and type III collagens. The interstitial accumulation of type l (A–D) and type lll (E–H) collagens was assessed by immunofluorescent staining for wild‐type (WT) sham‐operated (A and E), VASH1^+/−^ sham‐operated (B and F), WT unilateral ureteral obstruction (UUO) (C and G) and VASH1^+/−^ UUO (D and H) mice. The interstitial accumulation of collagens in the obstructed kidneys (OBK) was significantly exacerbated in the VASH1^+/−^ UUO (D and H) compared with the WT UUO (C and G) mice. The computer image analysis further confirmed the exacerbation of interstitial collagen accumulation in the OBK of the VASH1^+/−^ UUO mice (I and J). *P <**0.05 versus WT sham‐operated. ^#^*P *<**0.05 versus VASH1^+/−^ sham‐operated. ^$^*P *<**0.05 versus WT UUO. *n *=**5 for each group. Each column shows the means ± SEM.

We next examined the cortical interstitial accumulation of type l and type lll collagens. The immunoreactivity for interstitial type l and type lll collagens was not increased in the kidneys of the sham‐operated VASH‐1^+/−^ mice (Fig. 3B and F) compared with the WT mice (Fig. 3A and E). The interstitial accumulation of interstitial collagens in the OBK was significantly exacerbated in the VASH‐1^+/−^ UUO group (Fig. 3D and H) compared with the WT‐UUO group (Fig. 3C and G), which was further confirmed by the quantitative analysis (Fig. 3I and J).

### Immunohistochemical analysis of the interstitial FSP‐1^+^ and αSMA^+^ cells

**Figure 4.**
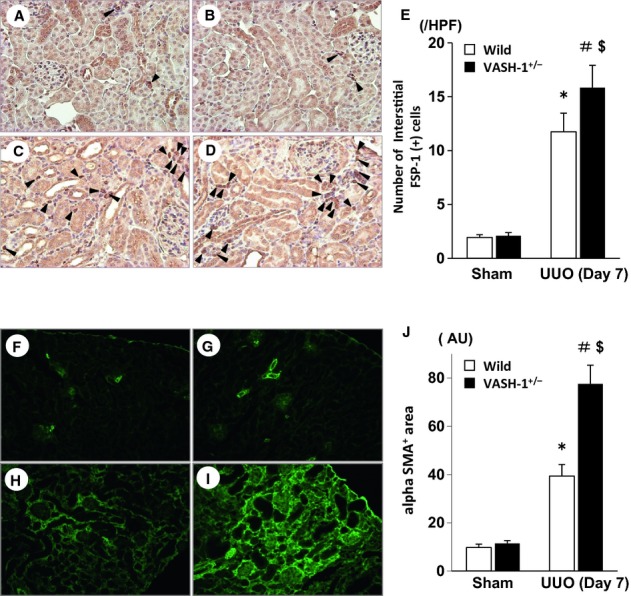
Immunohistochemical staining for FSP‐1 and *α*SMA. (A–D) The interstitial accumulation of FSP‐1^+^ fibroblasts (arrowheads) was assessed by an immunohistochemical analysis of wild‐type (WT) sham‐operated (A), VASH1^+/−^ sham‐operated (B), WT unilateral ureteral obstruction (UUO) (C) and VASH1^+/−^ UUO (D) mice. (E) The number of interstitial FSP‐1^+^ fibroblasts is shown. The quantitative analysis confirmed that there was a significant increase in interstitial FSP‐1^+^ fibroblasts in the obstructed kidneys (OBK) of the VASH‐1^+/−^ UUO mice. (F–I) The interstitial accumulation of *α*SMA^+^ myofibroblasts was assessed by immunofluorescence staining of the WT sham‐operated (F), VASH1^+/−^ sham‐operated (G), WT UUO (H) and VASH1^+/−^ UUO (I) mice. (J) The interstitial *α*SMA^+^ area relative to the total tubulointerstitial area was determined by a computer image analysis. The increase in the interstitial *α*SMA^+^ area in the OBK of VASH‐1^+/−^ mice (I) was significantly increased compared with that in the OBK of WT mice (H). *P <**0.05 versus WT sham‐operated. ^#^*P *<**0.05 versus VASH1^+/−^ sham‐operated. ^$^*P *<**0.05 versus WT UUO. *n *=**5 for each group. Each column shows the means ± SEM.

Renal interstitial fibrosis is associated with the accumulation of fibroblasts as well as their contractile subtype, myofibroblasts. Marked interstitial accumulation of FSP‐1^+^ fibroblasts was observed in the OBK of the WT mice (Fig. 4C). The interstitial accumulation of FSP‐1^+^ fibroblasts in the OBK was significantly increased in the VASH‐1^+/−^ UUO group (Fig. 4D) compared with the WT‐UUO group (Fig. 4C), as was confirmed by the quantitative analysis (Fig. 4E).

Marked interstitial accumulation of *α*SMA^+^ myofibroblasts was observed in the OBK of the WT mice (Fig. 4H). The number of interstitial *α*SMA^+^ myofibroblasts in the OBK was significantly increased in the VASH‐1^+/−^ UUO group (Fig. 4I) compared with the WT‐UUO group (Fig. 4H), as further indicated by the quantitative analysis (Fig. 4J).

### Immunohistochemical analysis of the interstitial infiltration of monocytes/macrophages

**Figure 5.**
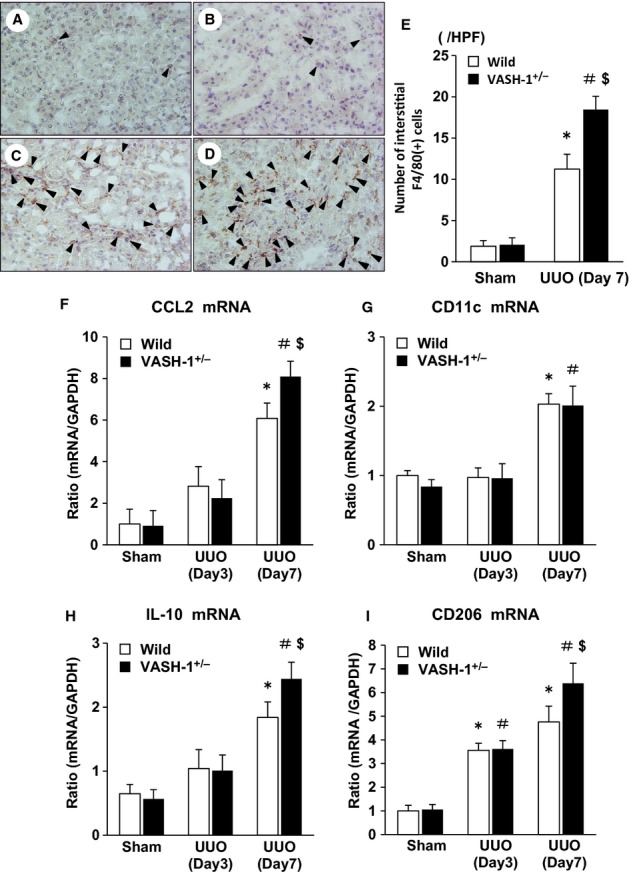
Exacerbated interstitial monocyte/macrophage infiltration in the VASH1^+/−^ unilateral ureteral obstruction (UUO) mice. (A–D) The interstitial accumulation of F4/80^+^ monocytes/macrophages (arrowheads) was assessed by performing an immunohistochemical analysis of the wild‐type (WT) sham‐operated (A), VASH1^+/−^ sham‐operated (B), WT UUO (C) and VASH1^+/−^ UUO (D) mice. (E) The number of interstitial F4/80^+^ monocytes/macrophages is shown. The quantitative analysis confirmed the significant exacerbation of the interstitial monocyte/macrophage infiltration in the obstructed kidneys (OBK) of VASH‐1^+/−^ mice. (F, G) The alterations in the renal mRNA levels of CCL2 and CD11c (real‐time PCR). (F) The UUO‐induced increase in the renal levels of CCL2 mRNA was significantly exacerbated in the VASH‐1^+/−^ mice compared with the WT mice. (G) The mRNA levels of CD11c in the OBK of WT and VASH‐1^+/−^ mice were increased similarly to those in the sham‐operated mice. (H, I) The alterations in the renal mRNA levels of IL‐10 and CD206 (real‐time PCR). (H) The IL‐10 mRNA levels were markedly elevated in the OBK of VASH‐1^+/−^ mice compared with those in the WT‐UUO group. (I) The CD206 mRNA levels were significantly elevated in the OBK of WT mice, and were further increased in the OBK of VASH‐1^+/−^ mice. *P <**0.05 versus WT sham‐operated. ^#^*P *<**0.05 versus VASH1^+/−^ sham‐operated. ^$^*P *<**0.05 versus WT UUO. *n *=**5 for each group. Each column shows the means ± SEM.

We next examined the interstitial infiltration of monocytes/macrophages in the OBK by performing immunohistochemical staining for F4/80. Although a very small number of interstitial F4/80^+^ monocytes/macrophages were observed in the sham‐operated WT or VASH‐1^+/−^ mice (Fig. 5A and B), marked interstitial accumulation was observed in the OBK of the WT mice (Fig. 5C) on Day 7. The interstitial accumulation of monocytes/macrophages in the OBK was significantly exacerbated in the VASH1^+/−^ UUO group (Fig. 5D) compared with the WT‐UUO group (Fig. 5C), as was confirmed by the quantitative analysis (Fig. 5E).

### VASH‐1 deficiency enhances the renal inflammatory reaction after UUO

We next examined the expression of pro‐inflammatory cytokines, monocyte chemoattractant protein‐1 (MCP‐1/CCL2), and CD11c (M1‐type monocyte/macrophage markers), and anti‐inflammatory cytokines, interleukin‐10 (IL‐10), and CD206 (an M2‐type monocyte/macrophage marker). The UUO‐induced increase in the renal levels of CCL2 mRNA was significantly exacerbated in the VASH‐1^+/−^ mice compared with the WT mice, as detected by a real‐time PCR analysis (Fig. 5F). The mRNA levels of CD11c in the OBK of the WT and VASH‐1^+/−^ mice were increased similarly to those in the sham‐operated mice (Fig. 5G). The IL‐10 mRNA levels were markedly elevated in the VASH‐1^+/−^ UUO group compared with the WT‐UUO group, as detected by a real‐time PCR analysis (Day7; Fig. 5H). The CD206 mRNA levels were significantly elevated in the OBK of the WT mice, and then they further increased in the OBK of the VASH‐1^+/−^ mice (Day7; Fig. 5I).

**Figure 6.**
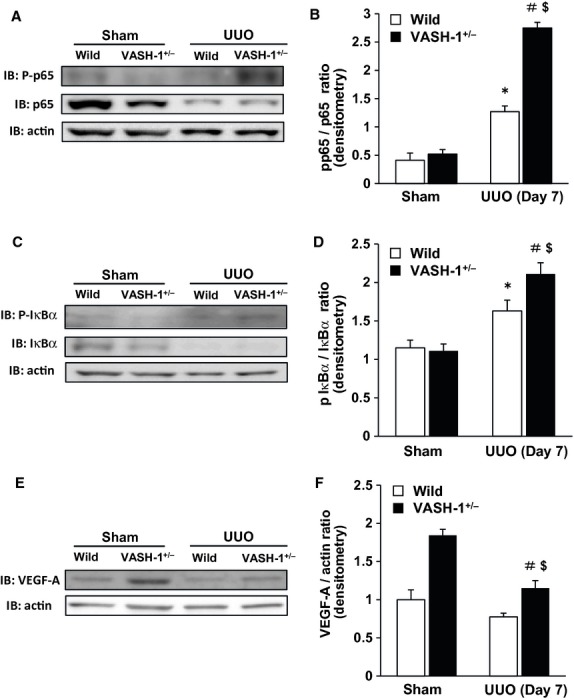
The increase in the renal level of phospho‐NF‐*κ*B/p65 and phospho‐I*κ*B*α* induced by unilateral ureteral obstruction (UUO) were enhanced in the VASH‐1^+/−^ mice. (A) A Western blot analysis of the phospho‐NF‐*κ*B/p65 level was performed. (A, B) The UUO‐induced increase in the renal levels of phospho‐NF‐*κ*B/p65 was significantly exacerbated in the VASH‐1^+/−^ mice compared with the wild‐type (WT) mice. (C, D) The UUO‐induced increase in the renal levels of phospho‐ I*κ*B*α* was significantly elevated in the VASH‐1^+/−^ mice compared with the WT mice. *P <**0.05 versus WT sham‐operated. ^#^*P *<**0.05 versus VASH1^+/−^ sham‐operated. ^$^*P *<**0.05 versus WT UUO. (E, F) The results of the Western blot analysis of VEGF‐A. The VEGF‐A protein levels were significantly increased in the VASH1^+/−^ sham‐operated mice compared with the WT sham‐operated mice. The VEGF‐A protein levels were significantly decreased in the VASH1^+/−^ UUO mice compared with the VASH1^+/−^ sham‐operated mice, but were significantly higher than those in the WT UUO mice. ^#^*P *<**0.05 versus VASH1^+/−^ sham‐operated. ^$^*P *<**0.05 versus WT UUO. *n *=**5 for each group. Each column shows the means ± SEM.

The UUO‐induced increase in the renal levels of phospho‐NF‐*κ*B/p65 was significantly exacerbated in the VASH‐1^+/−^ mice compared with the WT mice (Fig. 6A and B). The UUO‐induced increase in the renal levels of phospho‐I*κ*B*α* was significantly exacerbated in the VASH‐1^+/−^ mice compared with the WT mice (Fig. 6C and D).

### The role of VASH‐1 deficiency on PTC rarefaction and VEGF‐A expression

The renal levels of VEGF‐A were significantly increased in the VASH‐1^+/−^ sham‐operated mice compared with the WT sham‐operated mice (Fig. 6E and F). The VEGF‐A protein levels were significantly decreased in the VASH‐1^+/−^ UUO mice compared with the VASH‐1^+/−^ sham‐operated mice. The VEGF‐A levels in the OBK of VASH‐1 mice were mildly elevated compared with those in the WT mice (Fig. 6F).

**Figure 7.**
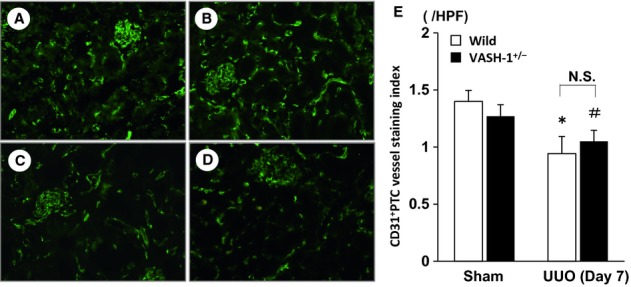
Immunofluorescent staining of CD31^+^ peritubular capillaries (PTC). The CD31^+^ PTC were assessed by immunofluorescent staining in the wild‐type (WT) sham‐operated (A), VASH1^+/−^ sham‐operated (B), WT unilateral ureteral obstruction (UUO) (C) and VASH1^+/−^ UUO (D) mice. (E) There were no significant differences in the densities of CD31^+^ PTC between the WT sham‐operated and VASH1^+/−^ sham‐operated mice. The PTC density in the WT UUO mice was significantly decreased compared with the WT sham‐operated mice, and compared with the VASH1^+/−^ sham‐operated mice, the PTC density was significantly decreased in the VASH1^+/−^ UUO mice. However, there were no significant differences in the density of the CD31^+^ PTC between the WT UUO and the VASH1^+/−^ UUO mice. *P < 0.05 versus WT sham‐operated control. ^#^*P* < 0.05 versus VASH1^+/−^ sham‐operated. *N* = 5 for each group. Each column shows the means ± SEM.

There were no significant differences in the density of CD31^+^ PTC between the WT sham‐operated and the VASH1^+/−^ sham‐operated mice (Fig. 7). The PTC density in the WT UUO mice was significantly decreased compared with that in the WT sham‐operated mice, but was not significantly different from that in the VASH1^+/−^ UUO mice.

### VASH‐1 deficiency enhances TGF‐β/Smad3 signaling in the UUO mice

**Figure 8.**
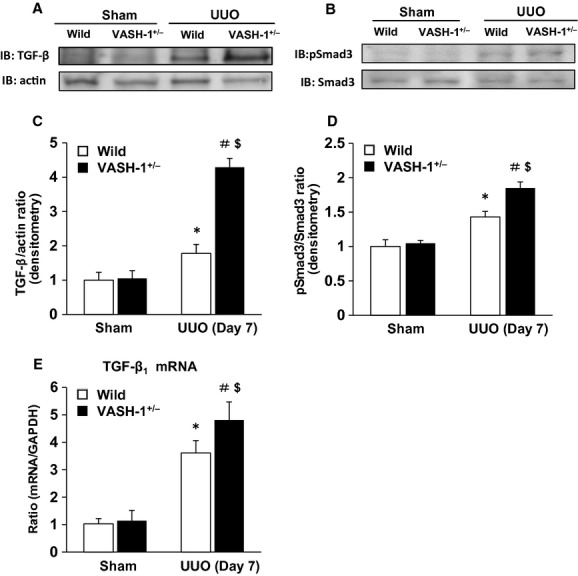
VASH‐1 deficiency enhanced the TGF‐*β*1/Smad3 signaling in the unilateral ureteral obstruction (UUO). (A, C, E) The UUO‐induced increase in the renal levels of TGF‐*β*1 was significantly exacerbated in the VASH‐1^+/−^ mice compared with the wild‐type (WT) mice as detected by immunoblots and real‐time PCR. (B, D) The UUO‐induced increase in the renal levels of phosphorylated Smad3 (normalized by the total Smad3 level) was significantly exacerbated in the VASH‐1^+/−^ mice compared with the WT mice. *P <**0.05 versus WT sham‐operated control. ^#^*P *<**0.05 versus VASH1^+/−^ sham‐operated. ^$^*P *<**0.05 versus WT UUO. *n *=**5 for each group. Each column shows the means ± SEM.

The UUO‐induced increase in the renal levels of TGF‐*β* was significantly exacerbated in the VASH‐1^+/−^ mice compared with the WT mice, as detected by immunoblotting and real‐time PCR (Fig. 8A, C and E). The UUO‐induced increase in the renal levels of phosphorylated Smad3 (normalized by the total Smad3) was significantly exacerbated in the VASH‐1^+/−^ mice compared with that in the WT mice (Fig. 8B and D).

### VASH‐1 deficiency exacerbates the TGF‐β1‐induced Smad3 signaling and matrix synthesis in vitro

**Figure 9.**
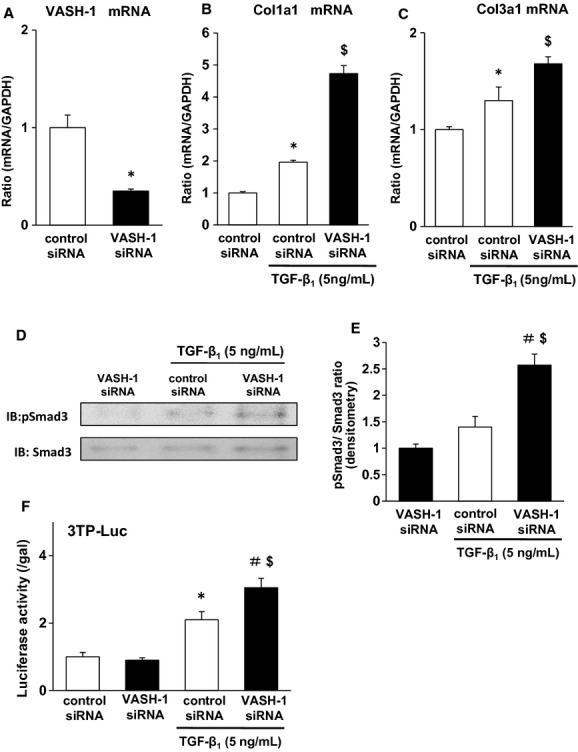
VASH1 deficiency exacerbated the TGF‐*β*1‐induced Smad3 signaling and matrix synthesis in cultured renal fibroblasts (NRK‐49F). (A) VASH‐1 was effectively inhibited by siRNA for VASH‐1. Real‐time PCR showed the knockdown of VASH‐1 mRNA by rat VASH‐1 siRNA in the NRK‐49F cells. (B, C) VASH‐1 siRNA treatment significantly upregulated the TGF‐*β*1‐induced synthesis of type I and III collagen mRNAs compared with the control siRNA. (D, E) VASH‐1 siRNA treatment significantly upregulated the TGF‐*β*1‐induced Smad3 phosphorylation compared with the control siRNA. (F) The Smad3‐responsive promoter assay (Luciferase assay). The cells were transiently transfected with a 3TP–Luc reporter plasmid (500 ng) and pCMV‐*β*‐gal (50 ng), and then were treated with TGF‐*β*1 (5 ng/mL) for 24 h. The cells were lysed, and the luciferase activity and *β*‐galactosidase (*β*‐gal) activity were measured and expressed as the ratio of luciferase to *β*‐gal activity. The results are shown as the means ± SEM of data from at least three separate experiments, each performed with triplicate samples. VASH‐1 siRNA treatment significantly up‐regulated the TGF‐*β*1‐induced Smad3‐dependent promoter activity compared with the control siRNA. *P <**0.05 versus siRNA (Control). ^#^*P *<**0.05 versus siRNA (VASH‐1). ^$^*P *<**0.05 versus siRNA (Control)/TGF‐*β*1. Each column shows the means ± SEM.

We next examined the effects of VASH‐1 knockdown in cultured rat renal fibroblasts (NRK‐49F). The VASH‐1 mRNA levels were decreased by VASH‐1 siRNA down to 35% of the level in the control (Fig. 9A). VASH‐1 siRNA treatment significantly upregulated the TGF‐*β*1‐induced synthesis of type I and type III collagen mRNAs compared with the control siRNA (Fig. 9B and C).

VASH‐1 siRNA treatment also significantly upregulated the TGF‐*β*1‐induced phosphorylation of Smad3 compared with the control siRNA (Fig. 9D and E). The enhanced Smad3‐mediated signaling in response to TGF‐*β*1 resulting from the VASH‐1 knockdown was further confirmed by a Smad3‐responsive reporter assay, where VASH‐1 siRNA treatment significantly upregulated the TGF‐*β*1‐induced Smad3‐dependent promoter activity compared with the control siRNA (Fig 9F).

## Discussion

The induction of UUO is followed by inflammatory cell infiltration, proliferation, and apoptosis of tubular epithelial cells, tubular atrophy and the accumulation of fibroblasts, resulting in interstitial fibrosis (Bascands and Schanstra 2005).

We previously reported the therapeutic effects of VASH‐1 overexpression in two mouse models of diabetic nephropathy (Nasu et al. 2009; Saito et al. 2011). However, the biological roles of VASH‐1 in the context of chronic tubulointerstitial injuries remain unclear. In the present study, we evaluated the functional role of endogenous VASH‐1 in the renal tubulointerstitial alterations in a mouse UUO model utilizing VASH‐1 heterozygous knockout mice. VASH‐1, an endogenous inhibitor of angiogenesis, was initially identified in a microarray analysis that assessed genes upregulated in endothelial cells by VEGF‐A (Watanabe et al. 2004). However, our recent studies have shown that it is expressed in various cell types, such as mesangial cells (Nasu et al. 2009), inflammatory cells (Miyake et al. 2009) and podocytes (Saito et al. 2011).

A previous report demonstrated that homozygous VASH‐1 knockout mice did not exhibit any significant changes in the vascular architecture at the steady state, but persistent subcutaneous angiogenesis was observed at the termination zone in VASH‐1 knockout mice (Kimura et al. 2009). The renal levels of VASH‐1 mRNA in the sham‐operated group were significantly lower in the VASH‐1^+/−^ mice compared with the WT littermates, and similar results were observed in the UUO group, suggesting that there was efficient suppression of endogenous VASH‐1 in our animals. The renal morphology was not altered in the sham‐operated VASH‐1^+/−^ mice, similar to the previous report (Kimura et al. 2009). The VASH‐1^+/−^ mice exhibited enhanced renal interstitial fibrosis and the accumulation of interstitial collagens in the OBK compared with their WT littermates, indicating the potential suppressive role of endogenous VASH‐1 on interstitial fibrosis. These results are consistent with the reported anti‐fibrotic effects of VASH‐1 in the experimental pulmonary fibrosis (Wang et al. 2010) and diabetic nephropathy models (Nasu et al. 2009; Saito et al. 2011).

Previous reports have demonstrated that Smad3, a key signaling intermediate downstream of the TGF‐*β* receptors, is a key molecule mediating TGF‐*β*_1_‐induced renal fibrosis in the UUO model. Under disease conditions, Smad2 and Smad3 are highly activated, while Smad7, the inhibitory Smad family member, is degraded through the ubiquitin proteasome system (Kavsak et al. 2000; Ebisawa et al. 2001). We previously reported that treatment with recombinant VASH‐1 resulted in the suppression of the increase of protein levels for TGF‐*β* induced by high glucose in cultured mesangial cells (Nasu et al. 2009). Although experimental models are different, direct inhibitory effect of Vasohibin‐1 on the synthesis of TGF‐*β* might be associated with the exacerbation of interstitial fibrosis in the OBK of the VASH‐1^+/−^ mice. VASH‐1^+/−^ mice exhibited enhanced renal interstitial fibrosis, interstitial accumulation of fibroblasts and myofibroblasts in the OBK in parallel with the significant increase in the level of TGF‐*β* and the phosphorylation of Smad3 compared with the WT littermates. These results suggest that physiological levels of VASH‐1 regulate TGF‐*β*_1_ Smad3‐dependent pro‐fibrotic signaling, which is associated with its potential anti‐fibrotic effects. The precise mechanism for endogenous VASH‐1 in regulating the increase of TGF‐*β* and its pro‐fibrotic effects remains to be elucidated.

Recent findings demonstrated the importance of the macrophage phenotype in determining the status of inflammation under pathological conditions. Classically activated M1 macrophages enhance inflammation, whereas alternatively activated M2 macrophages secrete anti‐inflammatory cytokines or profibrotic cytokines and play roles in tissue remodeling, wound healing, and UUO (Mosser 2003; Martinez et al. 2008; Braga et al. 2012; Yang et al. 2013). Significant exacerbation of the interstitial accumulation of monocytes/macrophages in the OBK was observed in the VASH‐1^+/−^ mice, consistent with our previous findings showing the anti‐inflammatory effects of VASH‐1 in models of diabetic nephropathy (Nasu et al. 2009; Saito et al. 2011). In the present study, the renal level of CCL2 mRNA in the OBK was further increased in the VASH‐1^+/−^ mice, supporting the accelerated inflammatory response in the absence of VASH‐1. Interestingly, the renal levels of CD11c (a cell surface marker for M1 macrophages) were similarly elevated in the OBK of the WT and VASH‐1^+/−^ mice, suggesting that there are qualitative alterations of M1 macrophages in the VASH‐1^+/−^ mice.

The levels of IL‐10, an anti‐inflammatory cytokine secreted by M2 macrophages, and CD206 (a cell surface marker of M2 macrophages) were elevated in the OBK of the WT mice compared with the sham‐operated controls, and were further increased in the OBK of the VASH‐1^+/−^ mice. A recent report by Wang Y et al. demonstrated the involvement of M2 macrophages in promoting renal interstitial fibrosis via the induction of the monocyte‐fibroblast transition (Yang et al. 2013). Therefore, the enhanced CD206 mRNA levels in the OBK of the VASH‐1^+/−^ mice might be associated with exacerbated renal interstitial fibrosis.

The NF‐*κ*B transcription factor plays an important role in proinflammatory signaling pathways, and is activated in human disease and experimental kidney disorders including UUO. The level of phosphorylated p65 as well as p‐I*κ*B*α* was elevated in the OBK of WT mice, and was further elevated in the VASH‐1^+/−^ mice. Collectively, the VASH‐1^+/−^ mice exhibited enhanced activation of the NF‐*κ*B signaling pathway, thus potentially leading to accelerated interstitial inflammatory cell infiltration.

The enhanced inflammatory alterations in the OBK of the VASH‐1^+/−^ mice observed in the present study might be associated with the role of VASH‐1 in enhancing the maintenance of endothelial cells by strengthening their resistance to various types of stress (Miyashita et al. 2012). In addition, a therapeutic effect of VASH‐1 on the formation of arterial neointima in association with the inhibitory effects on adventitial macrophage infiltration has been reported (Yamashita et al. 2006). As macrophages secrete TGF‐*β*1 (Leonarduzzi et al. 1997), the observed deteriorated inflammatory alterations in the OBK of the VASH‐1^+/−^ mice might have contributed to the increase of TGF‐*β* levels in association exacerbation of interstitial fibrosis.

To further analyze the mechanisms involved in the accelerated renal interstitial fibrosis in mice lacking VASH‐1, we next performed an in vitro study using normal rat kidney fibroblasts (NRK‐49F). Smad3‐binding elements are located in the promoter region of most of the collagen genes (Vindevoghel et al. 1998; Chen et al. 1999; Verrecchia et al. 2001), and thus, the TGF‐*β*1‐induced expression of collagen is mostly Smad3‐dependent. VASH‐1 siRNA treatment resulted in enhanced TGF‐*β*1/Smad3 signaling including the phosphorylation of Smad3, enhanced promoter activities, and the Smad3‐dependent synthesis of ECM molecules in the NRK‐49F cells. As recently reported by LeBleu et al. (2013), the renal residential fibroblasts serve as the main source of myofibroblasts and in turn promote the production of interstitial collagens, leading to interstitial fibrosis in renal diseases. Endogenous VASH‐1 may play a role in regulating the renal interstitial fibrosis driven by the resident fibroblasts.

In addition to TGF‐*β*1, many pathogenic mediators such as angiotensin‐II and advanced glycation end products can activate the Smad pathway via both TGF‐*β*‐dependent and independent mechanisms (Li et al. 2004; Wang et al. 2006; Chung et al. 2010). Smads interact with other signaling pathways, such as the MAPK and NF‐*κ*B pathways, to positively or negatively regulate renal inflammation and fibrosis (Wang et al. 2005a; Lan and Chung 2011). Both enhanced TGF‐*β*/Smad‐mediated signaling and activation of NF‐*κ*B were observed in the VASH‐1 deficient UUO mice. These pathways might have synergistically led to enhanced renal interstitial fibrosis.

Vascular endothelial growth factor (VEGF)‐A promotes angiogenesis (Ferrara 2000) and also induces vascular permeability, thus leading to inflammation (Dvorak et al. 1995). Previous reports have demonstrated that treatment with recombinant VEGF‐A resulted in reduction of interstitial fibrosis in association with preservation of PTC and stabilization of renal function in the rat remnant kidney model (Kang et al. 2001). Similarly, treatment with VEGF‐A resulted in the suppression of interstitial fibrosis as well as the levels of TGF‐*β*1 in a mouse UUO model at Day 3 or 7 following induction of UUO (Lian et al. 2011). These previous reports demonstrate the potential therapeutic effects of VEGF‐A in regulating renal interstitial fibrosis. In the present study, the VEGF‐A levels in the OBK of VASH‐1^+/−^ mice were significantly elevated compared with those in the WT mice. However, interstitial fibrosis as well as interstitial inflammation in the OBK of the VASH‐1^+/−^ mice was significantly deteriorated. Our results may suggest that slight elevation of renal VEGF‐A levels is not sufficient to overcome tubulointerstitial injuries, but exogenous administration of larger amount of VEGF‐A would be required for the therapeutic effects. In addition, slight increase of the endogenous VEGF‐A levels might rather promote inflammation, resulting in the deterioration of renal tubulointerstitial injuries considering the known association of VEGF‐A and inflammation (Dvorak et al. 1995).

With regard to the PTC density in the OBK, there were no significant differences between the VASH‐1^+/−^ and the WT mice in spite of the slight elevation of VEGF‐A levels in the VASH‐1^+/−^ mice. On the other hand, inflammation was not observed in the kidneys of the VASH‐1^+/−^ mice, in spite of the elevated VEGF‐A levels, suggesting the involvement of distinct anti‐inflammatory factors in the sham‐operated mice.

There are several limitations associated with the present study. Although we evaluated the regulatory role of endogenous VASH‐1 on the tubulointerstitial alterations induced by UUO, an evaluation in other models of renal fibrosis may be warranted. Second, the number of VASH‐1^−/−^ mice obtained through the mating of VASH‐1^+/−^ mice was limited for an unknown reason, and we could not evaluate the impact of homozygous VASH‐1 deficiency on renal interstitial fibrosis. Thirdly, we used the whole heterozygous VASH‐1 KO mice in the present study, but the particular cell type responsible for the regulatory roles of VASH‐1 in the progression of UUO may require examinations employing cell type‐specific conditional VASH‐1 knockout mice in the future. In addition, we demonstrated the effects of VASH‐1 deficiency in regulating ECM synthesis in fibroblasts. The potential anti‐fibrotic effects of VASH‐1 mediated via its regulation of the EMT, EndMT, and pericyte dedifferentiation may need further investigation. Supporting the potential role of VASH‐1 in these processes, we previously observed a role for VASH‐1 in maintaining the epithelial phenotype of podocytes in a model of diabetic nephropathy in vitro and in vivo (Saito et al. 2011).

In summary, this study provides novel evidence of a protective role for endogenous VASH‐1 against tubulointerstitial alterations via its regulation of ECM production, renal inflammation and fibrosis, and also demonstrates the potential anti‐fibrotic effects of VASH‐1 on renal fibroblasts through its modulation of TGF‐*β*1 signaling. The results of this study suggest that targeting VASH‐1 may represent a specific therapeutic approach for the treatment of renal diseases associated with progressive tissue fibrosis.

## Conflict of Interest

Prof. Yohei Maeshima belongs to endowed department by Chugai pharmaceutical, MSD, Boehringer ingelheim and Kawanishi Holdings. Prof. Hitoshi Sugiyama belongs to endowed department by Baxter. Prof. Hirofumi Makino is a consultant for AbbVie, Astellas and Teijin, receives speaker honoraria from Astellas, Boehringer‐ingelheim, Chugai, Daiichi Sankyo, Dainippon Sumitomo, Kyowa Hakko Kirin, MSD, Novartis, Pfizer, Takeda, and Tanabe Mitsubishi, and receives grant support from Astellas, Boehringer‐ingelheim, Daiichi Sankyo, Dainippon Sumitomo, Kyowa Hakko Kirin, Mochida, MSD, Novartis, Novo Nordisk, Pfizer, Takeda, and Tanabe Mitsubishi.
